# *In vitro* Antiproliferative and Apoptosis Inducing Effect of *Allium atroviolaceum* Bulb Extract on Breast, Cervical, and Liver Cancer Cells

**DOI:** 10.3389/fphar.2017.00005

**Published:** 2017-01-31

**Authors:** Somayeh Khazaei, Norhaizan M. Esa, Vasudevan Ramachandran, Roslida A. Hamid, Ashok K. Pandurangan, Ali Etemad, Patimah Ismail

**Affiliations:** ^1^Department of Biomedical Science, Faculty of Medicine and Health Sciences, Universiti Putra MalaysiaSerdang, Malaysia; ^2^Department of Nutrition and Dietetics, Faculty of Medicine and Health Sciences, Universiti Putra MalaysiaSerdang, Malaysia; ^3^Malaysian Research Institute of Aging, Universiti Putra MalaysiaSerdang, Malaysia; ^4^Department of pharmacology, Faculty of Medicine, University of MalayaKuala Lumpur, Malaysia

**Keywords:** *Allium atroviolaceum*, bulb, apoptosis, caspase, gene expression

## Abstract

Natural products are considered potent sources for novel drug discovery and development. The multiple therapeutic effects of natural compounds in traditional medicine motivate us to evaluate the cytotoxic activity of bulb of *Allium atroviolaceum* in MCF7 and MDA-MB-231, HeLa and HepG2 cell lines. The bulb methanol extract of *A. atroviolaceum* was found to be an active cell proliferation inhibitor at the time and dose dependent manner. Determination of DNA content by flow cytometry demonstrated S and G2/M phase arrest of MCF-7 cell, correlated to *Cdk1* downregulation, S phase arrest in MDA-MB-231 which is p53 and *Cdk1*-dependent, sub-G0 cell cycle arrest in HeLa aligned with *Cdk1* downregulation, G0/G1, S, G2/M phase arrest in HepG2 which is p53-dependent. Apoptosis as the mechanism of cell death was confirmed by morphology study, caspases activity assay, as well as apoptosis related gene expression, *Bcl-2*. Caspase-8, -9, and -3 activity with downregulation of *Bcl-2* illustrated occurrence of both intrinsic and extrinsic pathways in MCF7, while caspase-3 and -8 activity revealed extrinsic pathway of apoptosis, although *Bcl-2* downregulated. In HeLa cells, the activity of caspase-9 and -3 and downregulation of *Bcl-2* shows intrinsic pathway or mitochondrial pathway, whereas HepG2 shows caspase independent apoptosis. Further, the combination of the extract with tamoxifen against MCF7 and MDA-MB-231 and combination with doxorubicin against HeLa and HeG2 demonstrated synergistic effect in most concentrations, suggests that the bulb of *A. atroviolaceum* may be useful for the treatment of cancer lonely or in combination with other drugs.

## Introduction

Cancer is one of the leading causes of death and plant derived drugs have been used for the treatment of cancers due to the existence of various components with pharmacological properties. However, many bioactive compounds display potent cytotoxicity, the discovery of safe and less toxic drug of plant origin is necessary to fight against the fatal disease (George et al., 2016). The toxicity of anticancer drugs to normal fast-growing cells limits their efficacy. Further, resistance of cancerous cells to a specific drug which initially suppressed them is a major problem of chemotherapy drugs. Hence, using several drugs in combination may be more effective (Harvey, 2008).

The loss of balance between cell proliferation and apoptosis is a hallmark that increases the failure of damaged cells to be eliminated through apoptosis. One essential strategy for cancer therapy is to activate apoptotic pathways in the tumor cells. Many natural products that have been found to be a potential source of novel anticancer drugs, exert their antitumor effects by inducing apoptosis (Elkady et al., 2012). Apoptosis is induced by extracellular or intracellular signals, which trigger the signaling cascade with characteristic including nuclear condensation and DNA fragmentation (Ahmad et al., 2012). Furthermore, the deregulations responsible for initiation and promotion of cancer signify 100s of genes or signaling cascades (Teiten et al., 2010).

Caspase, a highly specific class of cysteine proteases, is known to mediate a crucial stage of the apoptotic process (Pourhassanali et al., 2016). Abundant *in vitro* and *in vivo* experiments confirmed that disordered regulation of caspase activation is crucial to avoid cancer cell death (Olsson and Zhivotovsky, 2011). Moreover, there are several genes known to involve in apoptotic pathways including *Bcl-2*, *Cdk1*, and *p53*. Anti-apoptotic *Bcl-2* overexpression has been implicated in different carcinomas (Guo et al., 2014). The mechanism through which *Bcl-2* inhibits apoptosis is considered to involve the inhibition of caspase proteins (Shi et al., 2015). Cyclin-dependent kinase1 (*Cdk1*) controls cell cycle entry from G2 to the M phase (Chen et al., 2015). *Cdk1*, which is overexpressed and has enhanced kinase activity in many tumor types, is a potential target for cancer therapy (Matthess et al., 2014). The *p*53 was found to be necessary for cellular senescence induced by alteration of genes involving mitosis and chromosome segregation (Fan et al., 2016). The *p*53 gene is able to activate cell cycle checkpoints, DNA repair and apoptosis to maintain genomic stability (Ahmad et al., 2012). Alteration or loss of *p*53 is crucial to the development of most malignancies (Lohrum and Vousden, 2000).

*Allium*s are commonly valued for food and medicine and range from plants to weeds. Onions, garlic, leek, chives and Welsh onions are some of the most commonly valued *Allium*s (Uhl, 2000). They are rich sources of tumor-inhibiting properties including organosulfur compounds and flavonols that can block several stages of carcinogenesis, although the underlying mechanisms of action are generally unclear. The association between the consumption of *Allium* vegetables and the risk of cancer indicates lower risks for cancers of the stomach, colon, esophagus and, perhaps, breast (Sengupta et al., 2004).

In this study, crude bulb extracts of *Allium atroviolaceum* (BAA) were tested to investigate the anti-proliferation activity of cancer cells, such as human hormone-dependent breast cancer (MCF7), human hormone-independent breast cancer (MDA-MB-231), human cervical cancer (HeLa), and human liver cancer (HepG2); additionally, its effects toward normal cells (3T3) were monitored to discover any probable harmful effect on normal cells. The study was then carried out to reveal the mechanism of action.

## Materials and Methods

### Plant Materials

Harvesting and preparation of fresh plant materials occurred during July (2013) from a local garden in North Iran. The plant was compared with voucher specimen No. 720–722 deposited at the Faculty of Biology Herbarium, Islamic Azad University of Ghaemshahr, Iran. BAA was rinsed, air dried and ground into powder form. About 5 g of plant material was placed in a thimble filter (25 mm × 80 mm) and 70% methanol (150 ml) was poured into a round bottom extraction flask. Extract of BAA was obtained using Soxhlet (Electrothermal, Eng., Rochford, UK). After 6 h of extraction, solvent was removed under reduced pressure by rotary evaporator (Büchi Labortechnik AG, Flawil, Switzerland) at a temperature not exceeding 50°C and then the solvent was completely removed by VirTis^®^ BenchTop^TM^ K freeze dryer (SP Scientific, Gardiner, NY, USA) with a 30 mm vessel for about 24 h. The dry residue of methanol extract (1.94 g) was dissolved in dimethyl sulfoxide (DMSO) (Sigma-Aldrich, St. Louis, MO, USA) to obtain the stock solution (1000 μg/ml).

### Cell Culture

MCF7 (human hormone-dependent breast cancer cell line; ATCC HTB-22), MDA-MB-231 (human non-hormone-dependent breast cancer cell line; ATCC HTB-26), HeLa (line; ATCC CCL-2), HepG2 (human hepatocellular cancer cell line; ATCC HB-8065), and 3T3 (mouse embryo fibroblast; ATCC CRL-1658) were obtained from American Type Culture Collection (Manassas, VA, USA). Cells were routinely maintained by culturing in RPMI-1640 medium (Sigma-Aldrich, Steinheim, Germany), supplemented with 10% fetal bovine serum (Sigma-Aldrich, Steinheim, Germany) and 100 IU/ml penicillin Streptomycin (Sigma-Aldrich, Steinheim, Germany). Cells were incubated in a direct heat humidified incubator (IR censored CO_2_ incubator) with 5% CO_2_ at 37°C.

### Cytotoxicity Assay

Cytotoxicity study was performed using MTT assay (Sigma-Aldrich, St. Louis, MO, USA). The cells (100 μl) were seeded in the 96 wells plate at a density of 1 × 10^6^ cells/ml and treated with various concentrations (1.56, 3.12, 6.25, 12.5, 25, 50, 100 μg/ml) of BAA following 24 h incubation. After 24, 48 and 72 h, 20 μg/ml of MTT was added and the cells were incubated for a further 4 h at 37°C. Thereafter, 100 μl of DMSO was added to each well and following incubation at room temperature for 15 min, the optical density of the formazan solution in each well was measured at 570 nm using FLUOstar Omega microplate reader (BMG Labtech, Ortenberg, Germany). The RPMI-1640 media, lacking any extract, was used as a negative control, while tamoxifen (TAM) (used for MCF7 and MDA-MB 231) and doxorubicin (DOX) (used for HeLa and HepG2) were the positive controls. In addition, to show that the BAA extract is safe for normal cells, the inhibitory effects of BAA were further evaluated on normal 3T3 cell line. Screening of the extracts on the five respective cell lines was done in triplicate with cell viability having been calculated by the following equation:

% Cytotoxicity = Absorbance of treated cells/Absorbance of negative control × 100. The 50% of growth inhibition concentration (IC_50_) was calculated from a plotted dose-response curve.

### Acridine Orange (AO) and Propidium Iodide (PI) Double Staining

All the cell lines were adjusted to 10^6^ cells/ml prior to treatment with BAA extract at the IC_50_ concentration in 25 cm^2^ tissue culture flasks and later incubated at 37°C for 24, 48, and 72 h. Thereafter, the cells were stained with 20 μl of AO/PI mixture, prepared by adding AO (10 μg/ml) (Sigma-Aldrich, St. Louis, MO, USA) and PI (10 μg/ml) (Sigma-Aldrich, St. Louis, MO, USA) to give a mixture of 1:1 (v/v) ratio dissolved in PBS. The mixture was visualized under Leica fluorescence microscope DM 2500 (Leica Microsystem, Wetzlar, Germany) with 100x magnification. Images were captured using an Alpha Imager (AlphaInnotech, San Leandro, CA, USA). Untreated cells were used as the negative control.

### Cell Cycle Distribution Analysis

The cells were seeded into 25 cm^2^ culture flasks at a density of 10^6^ cells/ml and allowed to adhere overnight. After treatment with the concentration of IC_25_, IC_50_ and IC_75_ (The concentration of BAA that induced 25, 50, and 75% growth inhibition) for 24, 48 and 72 h, the treated and untreated (negative control) cells were collected and centrifuged at 1,000 rpm for 10 min, and the pellet washed with cold PBS. Cells were re-suspended in 500 μl PBS and then fixed in 70% cold ethanol for at least 2 h at -20°C. After washing with PBS and centrifuge, the cells were incubated with a mixture of 500 μl PI/RNase (400 μl PI and 100 μl Ribonuclease A). Stained cells were incubated at room temperature in the dark for 30 min before being analyzed. Cell cycle profile was determined using a BD LSRFortessa^TM^ Cell Analyzer (Becton Dickinson, San Diego, NJ, USA) for 10,000 events per sample. Data were expressed as percentages of cells compared to the untreated control population, using analytical software BD FACSDiva^TM^.

### Annexin V/PI Apoptosis Detection Assay

The mode of cell death was performed by Annexin V-FITC apoptosis detection kit (Sigma-Aldrich, St. Louis, MO, USA) analysis was described according to the manufacturer’s protocol. The cells were seeded in 25 cm^2^ culture flasks at a density of 10^6^ cells/ml and incubated for 24 h before treatment with BAA extracts of IC_25_, IC_50_, and IC_75_ concentrations over different incubation periods (24, 48, and 72 h). The treated and untreated (negative control) cells were harvested by centrifugation (1000 rpm for 5 min) and re-suspended in 1x binding buffer. To stain the cells, 5 μl of Annexin V-FITC and 10 μl of PI solution was added to each suspension and incubated for 10 min at room temperature in the dark.

Induction of cell death was measured using a BD LSRFortessa^TM^ Cell Analyzer (Becton Dickinson, San Diego, NJ, USA).

### Caspase-3, -8, and -9 Colorimetric Assays

Quantitative determination of human caspase-3, -8, and -9 was performed by Caspase-family Colorimetric Substrate Set Plus (Biovision, Milpitas, CA, USA) according to the manufacturer’s instructions. Cells (10^6^/ml) were incubated for 24 h before treatment with BAA extract in different concentrations (IC_25_, IC_50_, and IC_75_). The treated and untreated (negative control) cells were harvested and centrifuged for 5 min at 1000 rpm and the pellets were lysed by addition a cold lysis buffer and incubated on ice for 10 min. After the cells were centrifuged at 5000 rpm for 2 min, 50 μl of cell lysate was transferred to a microplate. Each reaction required 50 μl of 2x reaction buffer containing 10 Mm DTT. At the last step, 5 μl of caspase *p*-nitroaniline (pNA) substrate was added into each well and incubated at 37°C for 1–2 h. The cleavage of the peptide by the caspase released the chromophore pNA. The level of caspase enzymatic activity in the cell lysate was directly proportional to the color reaction. Development and absorbance of colored product was recorded on a FLUOstar Omega microplate reader (BMG Labtech, Ortenberg, Germany) at a wavelength of 405 nm.

### Gene Expression Analysis by qRT-PCR

RNA extraction was carried out utilizing an RNeasy Mini kit (Qiagen, Inc., Valencia, CA, USA) for all the cell lines, untreated (negative control) or treated with IC_25_, IC_50_, and IC_75_ of BAA extract for 24 h. The procedure was performed according to the manufacturer’s instructions. The cDNA was synthesized from purified RNA with an RT2 First Strand Kit (Qiagen, Inc., Valencia, CA, USA) according to the manufacturer’s guidelines as the template for RT-qPCR. Corbett Rotor-Gene 6000 (Qiagen, Inc., Valencia, CA, USA) was used to perform quantitative real-time reverse transcriptase PCR (qRT-PCR). A final volume of 25 μl pre-mix was prepared containing 12.5 μl of RT2 SYBR^®^ Green ROX^TM^ FAST mastermix (Qiagen, Inc., Valencia, CA, USA), 1 μl of primers (RT^2^ qPCR Primer Assays, Qiagen, Inc., Valencia, CA, USA), 1 μl of cDNA, and 10.5 μl RNase-free water to make the final volume. The following primer pairs for target genes and GAPDH were chosen from the Primer Bank website. *Bcl-2*: 5′-TAC CTG AAC CGG CAC CTG-3′ and 5′-GCC GTA CAG TTC CAC AAA GG-3′; *Cdk1*: 5′-GGGTCAGCTCGCTACTCAAC-3′ and 5′-AAGTTTTTGACGTGGGATGC-3′; *p53*: 5′-TGT GGA GTA TTT GGA TGA CA-3′ and 5′-GAA CAT GAG TTT TTT ATG GC-3′; GAPDH: 5′-TCCTGCACCA CCAACTGCTTAG-3′ and 5′-GGCATGGACTGTGG TCATGAGT-3′.

The default PCR conditions were as follows: the PCR plate was run at 95°C for 10 min to activate the enzyme, 40 cycles of 15 s at 95°C (denaturation) followed by 30 s at 60°C (annealing and synthesis). Finally, the dissociation curve was constructed immediately after the PCR run to check and verify results. In relative quantification all samples were normalized to a constantly expressed housekeeping mRNA (reference mRNA) GAPDH. Only one reference gene was used in the current study because of limitation for interpretations. Relative gene expression was calculated for each mRNA marker using the 2^-ΔΔCt^ method: 2^ΔΔCt^ = 2^Ct^ ^(treatedcells)-Ct(controlcells)^, where 2 = the amplification efficiency where the template doubles in each cycle during exponential amplification.

### Drug Combination

To assess the combination effect of BAA extracts in combination with tamoxifen (TAM) (used for MCF7 and MDA-MB 231) and doxorubicin (DOX) (used for HeLa and HepG2), MTT assay was used as well. Briefly, cells were seeded at 1 × 10^6^ cells/well in the 96 wells plate for 24 h, treated with a serial dilution of combined BAA extracts and TAM (Sigma-Aldrich, St. Louis, MO, USA) or DOX (Sigma-Aldrich, St. Louis, MO, USA) and incubated for an additional 24, 48, and 72 h. Untreated media was used as the negative control. The IC_50_ value of the combination was determined by MTT assay. The combination index (CI) was used to analyze the synergistic inhibitory effects of drug combinations, which was evaluated by compusyn software. The CI values <1, =1, and >1 represent a synergistic, additive and antagonistic effect, respectively (Chang et al., 2006).

### Statistical Analysis

Results were analyzed by *version 7* of *GraphPad Prism*, using one-way analysis of variance (ANOVA), and differences were considered statistically significant at the level of *p*-values ≤ 0.05.

## Results

### Growth Inhibition and Viability

**FIGURE 1:**
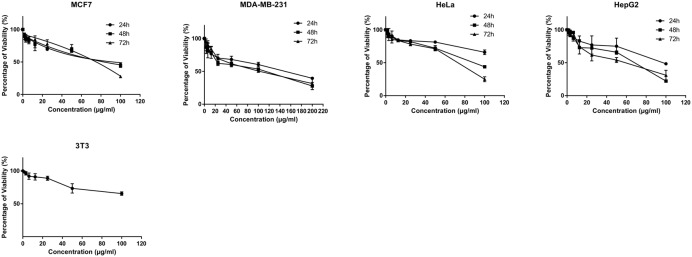
**Antiproliferative activity of BAA on MCF7, MDA-MB-231, HeLa, HepG2 and normal 3T3 cell lines.** Various concentrations of BAA extract showed cytotoxic effect on different cancer cell lines but not the normal cells at a time course of incubation. Data are presented as mean ± standard deviation of three independent experiments (*n* = 3).

**FIGURE 2:**
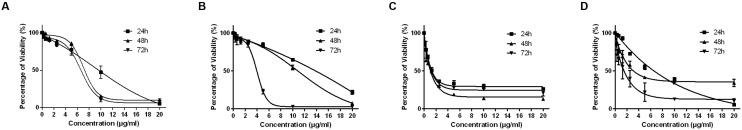
**The effect of TAM on exponentially growing MCF7 (A)** and MDA-MB-231 **(B)** and DOX on HeLa **(C)** and HepG2 **(D)** in a time- and dose-course manner. Values are calculated from three independent experiments (*n* = 3).

The MTT cytotoxicity assay exhibited different growth responses in MCF7, MDA-MB-231, HeLa, and HepG2 cells. The IC_50_ values after 24, 48, and 72 h of treatment were 91.5, 88, and 75.7 μg/ml for MCF-7 cells, 149, 114, and 101 μg/ml for MDA-MB-231, 154, 89.7, and 74.7 μg/ml for HeLa cells and 97, 70, and 58.7 μg/ml for HepG2, respectively. It is of note that the viability of untreated control cells remained high. Based on the IC_50_ values of BAA, the extract potency was in the order of HepG2 > MCF7 > HeLa > MDA-MB-231 at three periods of time. Moreover, the extract exhibited a selective anticancer effect with no cytotoxicity in normal 3T3 cells with the IC_50_ higher than 100 μg/ml (**Figure 1**). However, The IC_50_ of two commercial chemotherapy drugs, TAM (used against MCF7 and MDA) and DOX (used against HeLa and HepG2), demonstrated a capability of inducing cytotoxicity in cancer cell lines. The IC_50_ values for each cell line after 24, 48, and 72 h incubation were as follows: MCF7, 9.8, 7.2 and 6.7 μg/ml, MDA-MB 231, 13.6, 11, 3.8 μg/ml, HeLa, 1.4, 1.37, 1.27 μg/ml, and HepG2, 4.75, 3.47, 1.7 μg/ml, respectively (**Figure 2**).

#### Fluorescent Microscopy Study (AO/PI Staining)

**FIGURE 3:**
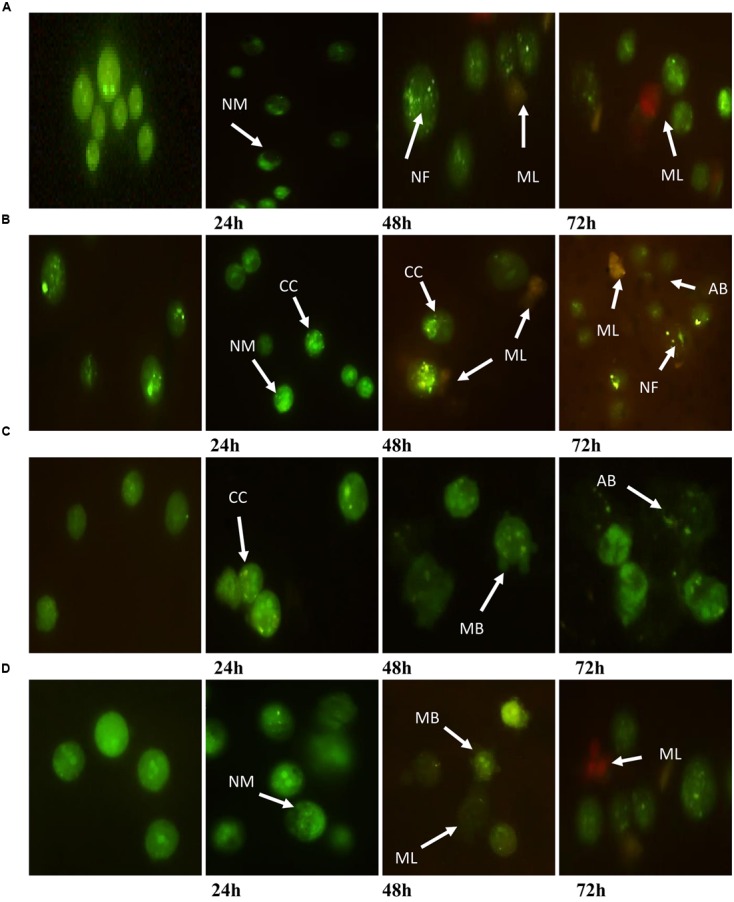
**Morphological changes observed in AO/PI stained (A)** MCF7, **(B)** MDA-MB-231, **(C)** HeLa, and **(D)** HepG2 cells. The figure from left to right are control (untreated) cells, cell treated for 24, 48, and 72 h for each cell line. Treated cells showed the typical characteristic of apoptosis such as nuclear margination (NM), chromatin condensation (CC), nuclear fragmentation (NF), membrane blebbing (MB), apoptotic bodies (AB), and membrane loose (ML).

A fluorescent microscopy study was undertaken to understand the mode of cell death. AO/PI staining revealed that after the treatment of MCF7 at 24 h, the cell showed typical morphological features of apoptosis, including nuclear margination and chromatin condensation. After 48 h, granulations in the nucleus were clearly observed. However, membrane loss occurred in cells at 48 and 72 h (**Figure 3A**). Moreover, MDA-MB-231 cells exhibited nuclear margination and chromatin condensation, which were major consequences of the apoptotic trigger, after treatment at 24 h, while the 48 and 72 h treatments represented both early (chromatin condensation, nuclear fragmentation) and late apoptosis (apoptotic bodies, membrane loose) features (**Figure 3B**). Conversely, HeLa cells treated for 24 h exhibited early apoptotic cells with chromatin condensation and nuclear margination stained with a bright-green color. Early apoptosis features were displayed after the treatment of cells for 48 h (membrane blebbing); however, nuclear fragmentation and apoptotic bodies were clearly observed at 72 h (**Figure 3C**). Furthermore, an observation of HepG2 after treatment found that the cells displayed green fluorescence with the appearance of nuclear margination (24 h) and membrane blebbing (48 h), which were considered as moderate apoptosis. In addition, green–orange fluorescence which illustrated the late stages of apoptosis appeared after 72 h of treatment. Furthermore, the presence of red color due to the binding of AO to denatured DNA was clearly seen (**Figure 3D**).

### The Effect on Cell Cycle Distribution

**FIGURE 4:**
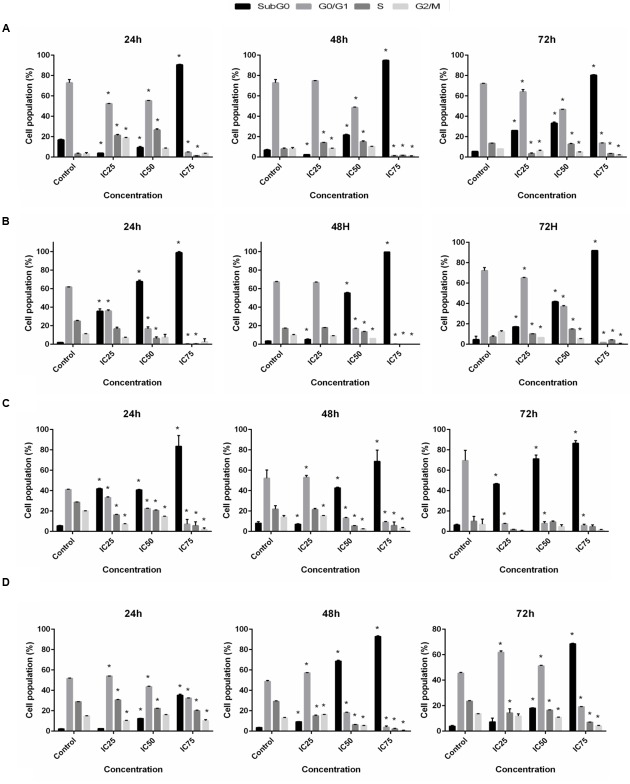
**Cell cycle distribution after BAA treatment in different cell lines.** Variation in the percentage of **(A)** MCF7, **(B)** MDA-MB-231, **(C)** HeLa, **(D)** HepG2 cells present in each phase of the cell cycle between untreated cells and cells exposed to different concentration of BAA for 24, 48, and 72 h. Data represent the mean ± SD (*n* = 3). ^∗^*p* < 0.05 compares the treated cell with control cell. IC_25_ and IC_75_ in MCF7 = 103 ± 2.64, 46.5 ± 2.12; MDA-MB-231 = 196.67 ± 5.77, 17.67 ± 2.53; HeLa = 98.33 ± 2.89, 43.67 ± 2.8; HepG2 = 99.33 ± 1.15, 14 ± 5.29, respectively.

Inhibition of proliferation was further examined by measuring cell cycle distribution. After the incubation of cells at 24, 48, and 72 h with three different concentrations (IC_25_, IC_50_, and IC_75_) of BAA, a significant increase in the cell population at the sub-G0 was observed when compared with the control (*p* ≤ 0.05) for all cell lines. The results also exhibited a lower proportion of MCF7, MDA-MB-231 and HeLa in the G0/G1 phase which illustrated there was no cell cycle arrest detected in G0/G1 phase in these cells. Conversely, treated MCF7 cells presented induced percentages of cells in the S phase by IC_25_ and IC_50_, and a noticeable G2/M arrest with IC_25_ at 24 h. At 48 h, the cell population in S phase increased by IC_25_ and IC_50_ (**Figure 4A**). Meanwhile, MDA-MB-231 cells revealed an induction in the S phase after treatment with IC_25_ and IC_50_ concentrations, compared to the control at 72 h (**Figure 4B**). HeLa cell distribution only in sub-G0 indicated the occurrence of apoptosis (**Figure 4C**). HepG2 cancer cells indicated a significant enhancement in cell proportion in the G0/G1 phase after treatment with IC_25_ of BAA at 48 h. Likewise, the cells treated with IC_25_ concentration of BAA showed a significant increase at 48 h. On the other hand, IC_25_ and IC_50_ of BAA significantly elevated the cells’ percentage in the G0/G1 phase after 72 h (**Figure 4D**). Taken together, BAA caused growth arrest in the G0/G1 phase of the cell cycle only in HepG2, but in MCF7, MDA-MB-231 and HeLa cells, the induction of cytotoxicity occurred through the mechanisms associated with apoptosis.

### Induction of Apoptosis

Since the proportion of cells with hypodiploid DNA content (or apoptotic cells) was considerably high in cell cycle, Annexin V-FITC/PI staining was examined by using flow cytometry to further investigate apoptosis induction by BAA over time and dose. The flow cytometric analysis of treated cells revealed a dramatic reduction in the percentage of viable cells from a dose and time-dependent aspect in all the studied cell lines. In MCF7 cells, early apoptosis significantly increased by all concentrations of BAA after 24 h, by IC_75_ after 48 h and by IC_50_ and IC_75_ after 72 h. Meanwhile, late apoptosis increased markedly in cells treated only with IC_50_ of BAA at 24 h and by IC_75_ at 48 h whereas BAA caused apoptosis induction with increasing concentration when compared to the untreated cells at 72 h. In contrast, the percentages of cells entering necrosis decreased by IC_25_ and IC_75_ at 24 h and by IC_50_ and IC_75_ of BAA at 72 h. The results of BAA extract effect on MDA-MB-231 showed the treated cells entered early apoptosis stage in IC_25_, IC_50_ and IC_75_ concentrations after 24 and 48 h. However, at 72 h, the proportion of early apoptotic cells decreased compared to 24 and 48 h; nevertheless, they were still higher than control cells. The percentages of cells entering late apoptosis and necrosis were not significant in treated cells at 24 h, while the cells presented in the late apoptosis stage considerably induced in IC_50_ and IC_75_ values at 48 h and all concentrations of BAA after 72 h. The percentages of apoptotic HeLa cells treated with BAA showed a significant dose-dependent increase in early apoptosis after treatment with all concentrations at 24 h, IC_75_ at 48 h and IC_50_ at 72 h. In contrast, the proportion of late apoptosis significantly increased only in IC_75_ at 24 h, IC_50_ and IC_75_ at 48 h and by all the concentrations of BAA after 72 h. Conversely, the necrotic cells were significantly reduced by IC_75_ at 24 h, but increased after 48 and 72 h. Treatment of HepG2 cells by BAA revealed entering into early apoptosis had been sharply enhanced by IC_75_ of BAA at 24 h and IC_50_ and IC_75_ of BAA at 48 and 72 h. The cell proportion present in late apoptosis was greatly raised by IC_25_ and IC_75_ of BAA at 24 h, IC_75_ at 48 h and IC_50_ and IC_75_ at 72 h. No significant effect was recorded in the percentages of necrotic cells in most of the concentrations (**Table 1**).

**Table 1:** Induction of apoptosis in different cell lines with no treatment and treatment with BAA at IC_25_, IC_50_, and IC_75_ for 24, 48, and 72 h.

MCF7									
**Test extract**	**Time**	***V***	***p*-value**	***E***	***p*-value**	***L***	***p*-value**	***N***	***p*-value**
Control	24 h	96.15 ± 0.07	-	0.15 ± 0.07	-	0.9 ± 0.14	-	2.8 ± 0.14	-
	48 h	98.8 ± 0.14	-	1.15 ± 0.7	-	0	-	0	-
	72 h	96	-	0.05 ± 0.07	-	0.6 ± 0.14	-	3.4 ± 0.14	-
BAA IC25	24 h	91.6 ± 0.56ˆ*	0.019	7.7 ± 1.13ˆ*	0.002	0.7 ± 0.56	0.99	0^∗^	0.033
	48 h	98.1 ± 0.14	1	1.75 ± 0.07	1	0.1	1	0	1
	72 h	90.85 ± 1.2	0.064	1.75 ± 0.21	0.649	4.7 ± 2.26	0.095	2.75 ± 1.2	0.73
BAA IC50	24 h	85.55 ± 1.34ˆ*	0.001	5.8 ± 0.42ˆ*	0.005	5.7 ± 2.12ˆ*	0.044	2.95 ± 1.2	0.99
	48 h	94 ± 0.28	0.948	4.9	0.986	1.05 ± 0.21	0.965	0	1
	72 h	64.45 ± 1.62ˆ*	0.00	16.3 ± 1.27ˆ*	0.001	19 ± 0.42ˆ*	0.00	0.3^∗^	0.024
BAA IC75	24 h	68.4 ± 0.84ˆ*	0.000	29.65 ± 0.77ˆ*	0.00	1.4 ± 0.56	0.97	0^∗^	0.033
	48 h	24.25 ± 18.17ˆ*	0.004	59.35 ± 22.55ˆ*	0.023	16.25 ± 4.59ˆ*	0.007	0.15 ± 0.21	0.553
	72 h	9.2 ± 1.83ˆ*	0.00	32.7 ± 2.47ˆ*	0.00	57.75 ± 0.91ˆ*	0.00	0.25 ± 0.21ˆ*	0.023

**MDA-MB231**									
Control	24 h	98.3 ± 0.14	-	1.4 ± 0.14	-	0.1	-	0.25 ± 0.07	-
	48 h	97.95 ± 0.07	-	1.85 ± 0.07	-	0.15 ± 0.07	-	0	-
	72 h	96.96 ± 1.5	-	0.76 ± 0.4	-	1.83 ± 0.8	-	0.46 ± 0.23	-
BAA IC25	24 h	94.65 ± 0.49	0.86	4.7 ± 1.13	0.492	0.2 ± 0.14	1	0.35 ± 0.49	0.976
	48 h	85.95 ± 0.21	0.85	13.5 ± 0.14ˆ*	0.043	0.55 ± 0.07	0.999	0	1
	72 h	71.2 ± 1.98ˆ*	0.00	0.76 ± 0.4ˆ*	0.015	20.25 ± 0.8ˆ*	0.00	0.46 ± 0.23ˆ*	0.015
BAA IC50	24 h	75.7 ± 0.28ˆ*	0.028	23.6 ± 0.28ˆ*	0.002	0.6 ±	0.997	0.1 ±	0.929
	48 h	26.75 ± 4.31ˆ*	0.00	61.75 ± 5.3ˆ*	0.00	11.5 ± 0.98ˆ*	0.092	0	1
	72 h	32.95 ± 0.63ˆ*	0.00	4.1 ± 0.42	0.13	61.5^∗^	0.00	1.4 ± 0.28ˆ*	0.046
BAA IC75	24 h	31 ± 9.33ˆ*	0.00	64.1 ± 4.1ˆ*	0.00	4.85 ± 5.16	0.375	0.05 ± 0.07	0.855
	48 h	8.1 ± 5.51ˆ*	0.00	55.1 ± 1.27ˆ*	0.00	32.75 ± 6.71ˆ*	0.002	4.1 ± 0.14ˆ*	0.00
	72 h	1 ± 0.56ˆ*	0.00	6.2 ± 1.83ˆ*	0.023	92.85 ± 2.33ˆ*	0.00	0	0.339

**HeLa**									
Control	24 h	94.45 ± 0.14	-	2.2 ± 0.21	-	0.95 ± 0.07	-	2.4 ± 0.42	-
	48 h	98.05 ± 0.14	-	1.3 ± 0.35	-	0.2	-	0.4 ± 0.14	-
	72 h	98.75	-	0.6 ± 0.21	-	0.6 ± 0.14	-	0.1	-
BAA IC25	24 h	77.15 ± 0.21ˆ*	0.00	17.95 ± 0.21ˆ*	0.002	2.4 ± 0.14	0.659	2.45 ± 0.21	0.981
	48 h	90.7 ± 0.21ˆ*	0.003	1.2 ± 0.14	0.998	2.25 ± 0.21	0.077	5.95 ± 0.14ˆ*	0.00
	72 h	84.6 ± 0.42ˆ*	0.00	0.3 ± 0.98	0.491	5.3 ± 0.56ˆ*	0.02	9.8 ± 0.14ˆ*	0.00
BAA IC50	24 h	58.4 ± 0.07ˆ*	0.00	33.55 ± 0.42ˆ*	0.00	5.05 ± 0.21	0.088	3.05 ± 0.21ˆ*	0.03
	48 h	65.5 ± 0.49ˆ*	0.00	0.2 ± 0.14	0.329	21.4 ± 0.07ˆ*	0.00	12.8 ± 0.35ˆ*	0.00
	72 h	68.4 ± 0.84ˆ*	0.00	16.25 ± 0.35ˆ*	0.00	15.1 ± 1.27ˆ*	0.00	0.25 ± 0.07	0.956
BAA IC75	24 h	7.7 ± 0.56ˆ*	0.00	69.65 ± 2.89ˆ*	0.00	22.4 ± 2.4ˆ*	0.00	0.25 ± 0.07ˆ*	0.00
	48 h	8.2 ± 0.05ˆ*	0.00	56 ± 1.61ˆ*	0.00	35.7 ± 0.91ˆ*	0.00	0.13 ± 0.85	0.253
	72 h	31.15 ± 0.63ˆ*	0.00	0.75 ± 0.07	0.863	51.85 ± 1.06ˆ*	0.00	16.2 ± 0.42ˆ*	0.00

**HepG2**									
Control	24 h	96.7 ± 2.4	-	3.2 ± 2.54	-	0.1 ± 0.14	-	0	-
	48 h	98.1 ± 0.98	-	1.25 ± 0.77	-	0.45 ± 0.21	-	0.25 ± 0.07	-
	72 h	92.8 ± 3.25	-	2.5 ± 1.27	-	4.45 ± 1.9	-	5.4 ± 0.07	-
BAA IC25	24 h	96.45 ± 0.21	0.997	1.7	0.726	0.7^∗^	0.016	1.15 ± 0.21ˆ*	0.002
	48 h	93.45 ± 0.49	0.195	6.2 ± 0.56	0.14	0.3	0.759	0.05 ± 0.07	0.098
	72 h	88 ± 1.13	0.154	5 ± 1.27	0.205	6.6 ± 0	0.42	0.4 ± 0	0.919
BAA IC50	24 h	91.2 ± 0.84	0.052	8.6 ± 0.84	0.06	0.15 ± 0.07	0.962	0.05 ± 0.07	0.971
	48 h	83.95 ± 2.19ˆ*	0.005	15.5 ± 2.12ˆ*	0.004	0.5	0.985	0.05 ± 0.07	0.098
	72 h	63.65 ± 0.35ˆ*	0.00	9.2 ± 0.14ˆ*	0.009	16 ± 0.42ˆ*	0.003	11.15 ± 0.21ˆ*	0.00
BAA IC75	24 h	37.4 ± 0.98ˆ*	0.00	62 ± 0.84ˆ*	0.00	0.6 ± 0.14ˆ*	0.031	0.05 ± 0.07	0.971
	48 h	23.95 ± 2.75ˆ*	0.00	74.6 ± 2.54ˆ*	0.00	1.35 ± 0.21ˆ*	0.013	0.1	0.209
	72 h	21.75 ± 0.21ˆ*	0.00	9.75 ± 0.91ˆ*	0.007	48.7 ± 1.55ˆ*	0.00	19.8 ± 0.42ˆ*	0.00

*Data are presented as the percentage of the cells in four sub-populations. V: viable cells, E: early apoptosis, L: late apoptosis, N: necrosis. Data represents the mean ± SD (n > 3). ^∗^p < 0.05.*

### Induction of Caspase Activity

**FIGURE 5:**
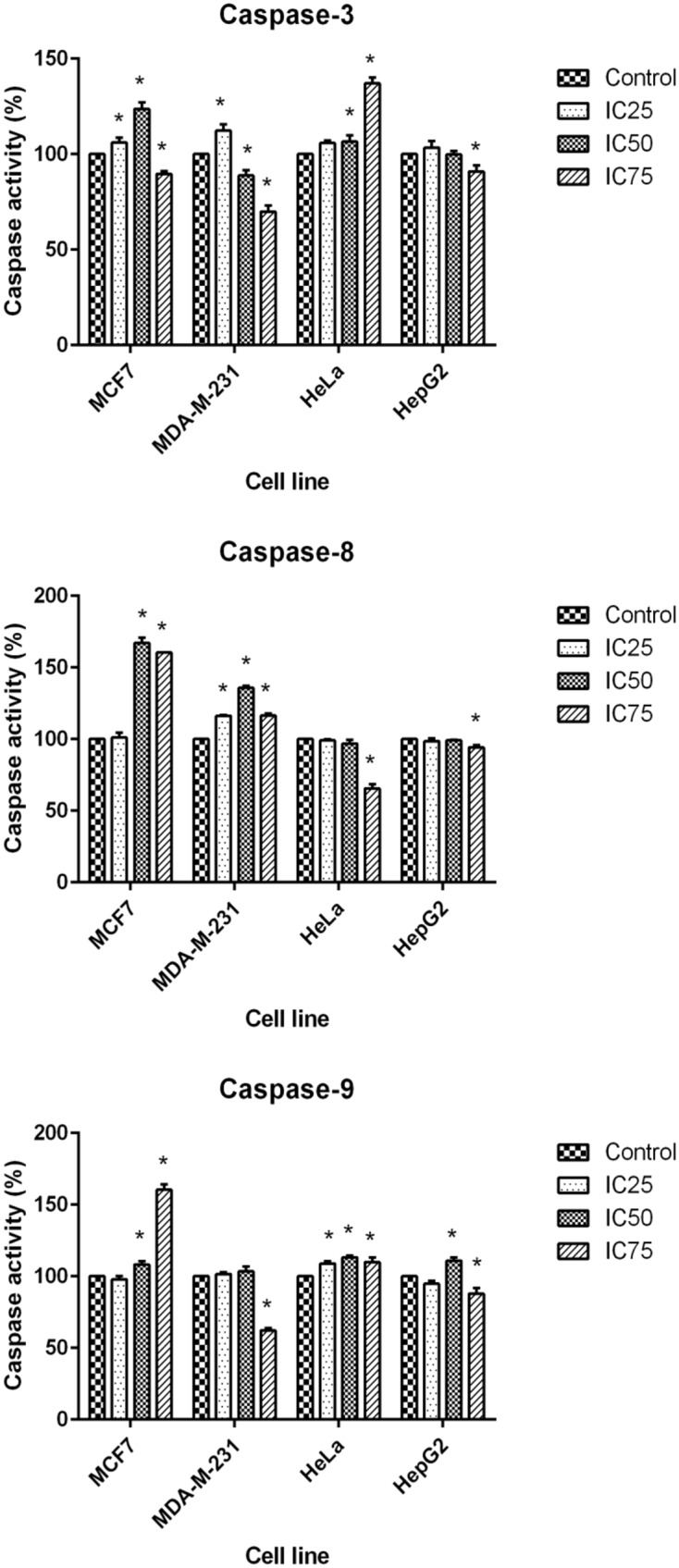
**Activity of caspase-3, -8, and -9 in MCF7, MDA-MB-231, HeLa, and HepG2 cells after 24 h treatment with IC_25_, IC_50_, and IC_75_ of BAA.** Results are expressed as the mean optical density (405 nm) ± SD (*n* = 3). ^∗^*p* < 0.05 compares the treated cell with control cell.

The final stage of apoptosis occurs through the activation of caspases (Soung et al., 2008) and discovering the activation pathway by cytotoxic agents may offer essential knowledge in modeling better treatment strategies (Tee et al., 2007). In the current study, the activation of caspase-3, -8, and -9 was investigated at 24 h for three concentrations of BAA extract. MCF7 cells, following exposure to BAA extract, showed significant elevation of caspase-3 activity in IC_25_ and IC_50_ concentrations, while MDA-MB-231 showed an early induction of caspase-3 activity only in a low concentration (IC_25_). The activity of caspase-3 in treated HeLa cells was raised by IC_50_ and IC_75_, whereas it was not significantly changed in the HepG2 cells observed, compared to the control cells. Moreover, the extract of BAA was able to trigger caspase-8 activity in both MCF7 and MDA-MB-231 cell at IC_50_. In contrast, low caspase-8 activity in HeLa and HepG2 cell lines showed that the extrinsic pathway might not be activated by BAA extract. Furthermore, caspase-9 activity increased in BAA treated MCF7 cells at IC_50_ and IC_75_ of BAA, while MDA-MB-231 treatment did not increase the activation of caspase-9. The results evaluated the potentially higher activity of caspase-9 when HeLa cells were exposed to all three concentrations of BAA, whereas HepG2 cell lines exposed to BAA extracts showed a significant enhancement of caspase-9 activity only in IC_50_ (**Figure 5**).

### Gene Expression Analysis of Apoptosis- and Cell Cycle-Related Genes Modulated by BAA

**FIGURE 6:**
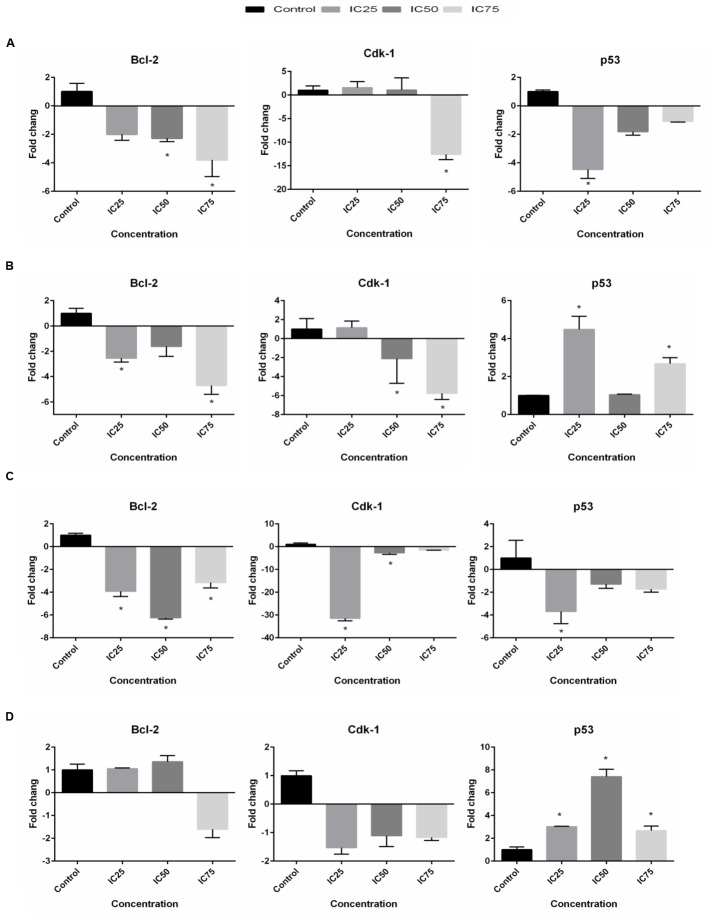
**BAA effects on *Bcl-2*, *Cdk1* and *p53* mRNA levels in (A)** MCF7, **(B)** MDA-MB-231, **(C)** HeLa, and **(D)** HepG2 cells after treatment with the IC_25_, IC_50_, and IC_75_ for 24 h. The relative quantification of the target gene by the delta–delta–Ct method was done using the Qiagen software. Data represent the mean ± SD (*n* = 3). ^∗^*p* < 0.05 compares the treated cell with control cell.

Quantitative real-time PCR was utilized to analyze mRNA levels of apoptotic- and cell cycle-related genes in the selected cells exposed to BAA at concentrations of IC_25_, IC_50_, and IC_75_ for 24 h. The expression of *Bcl-2* gene in the treated MCF7 cells was found to be dramatically downregulated 2.28- and 3.8-fold by IC_50_ and IC_75_, while IC_75_ had remarkably downregulated the *Cdk1* gene expression 12.6-fold (**Figure 6A**). Treatment of MDA-MB-231 with IC_25_ and IC_75_ downregulated the expression of *Bcl-2* 2.56- and 4.71-fold, respectively. A significant decrease in mRNA level of *Cdk1* was observed in MDA-MB cells after treatment with IC_50_ and IC_75_ 2.11- and 5.77-fold, respectively. On the other hand, treated MDA-MB-231 showed *p53* upregulation by BAA at IC_25_ and IC_75_ 4.48- and 2.67-fold, respectively (**Figure 6B**). BAA had, likewise, a strong impact on the expression of *Bcl-2* in HeLa cells, since it decreased the level of *Bcl-2* with IC_25_, IC_50_, and IC_75_ concentration 3.93-, 6.25-, and 3.16-fold, respectively. BAA also downregulated the expression of *Cdk1* in HeLa cells in IC_25_ and IC_50_ concentrations, 31.45- and 2.68-fold (**Figure 6C**). Expression levels of *Bcl-2* and *Cdk1* in HepG2 cells was not significant, while a significant upregulation occurred in *p53* expression in HepG2, exclusively during the treatment of IC_25_, IC_50,_ and IC_75_ by inducing significant 3.03-, 7.41-, and 2.67-fold upregulations in its levels, respectively (**Figure 6**).

### Detection of Drug–Drug Interactions

**FIGURE 7:**
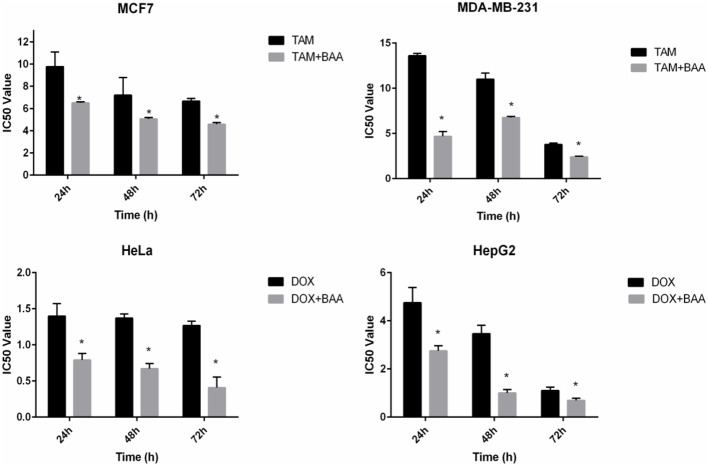
**Enhanced growth inhibitory effect of BAA-TAM or BAA–DOX combination on exponentially growing (A)** MCF7, **(B)** MDA-MB-231, **(C)** HeLa, and **(D)** HepG2. Values are calculated from three independent experiments. ^∗^*p* < 0.05 compare the combination of BAA-TAM or BAA–DOX with TAM or DOX alone.

In the current study the synergistic inhibitory effect of BAA and the conventional chemotherapeutic drug TAM and DOX were investigated. The IC_50_ values of co-treatment with BAA-TAM combination reduced to 6.5, 5.07, and 4.58 μg/ml in MCF7 and 4.7, 6.77, and 2.42 μg/ml in MDA-MB-231 after 24, 48, and 72 h, respectively. Moreover, the BAA-DOX combination reduced the IC_50_ to 0.79, 0.9, and 0.4 μg/ml in HeLa cells and 2.75, 1, and 0.69 μg/ml in HepG2, at 24, 48, and 72 h, respectively (**Figure 7**).

Drug combination effects were analyzed by the Chou and Talalay method to investigate whether the drugs are synergist, additive or antagonist. In this model, the CI values were calculated based on the gradient and the IC_12.5_, IC_25_, IC_50_, and IC_75_ values of each dose response curve (drug alone or in combination) (Zhao et al., 2004). CI values calculation was completed using the CompuSyn software (ComboSyn, Inc., Paramus, NJ, USA) (Zhao et al., 2014). In the MCF7 cell line, the CI was reduced in a dose-dependent manner at 24 h, although the lower concentrations (IC_12.5_ and IC_25_) produced an antagonistic effect, the higher concentration (IC_50_ and IC_75_) revealed a synergistic effect. In contrast, the CI indicated a synergistic effect in IC_25_ and IC_50_, while exhibiting an antagonistic effect in IC_12.5_ and IC_75_ at 48 h. At 72 h, the CI resulted in synergism only at IC_50_, but antagonism at IC_12.5_, IC_25_, and IC_75_. The calculated CI values in the BAA-TAM treatment of MDA-MB-231 showed the combinations were synergistic in medium and high dose levels at 24 h. At 48 h, except for the highest concentration (IC_75_) all other combinations had antagonistic effects. By contrast, the synergistic effects at 72 h were recorded at low dose levels (IC_12.5_) while all the other combinations resulted in antagonistic effects. In HeLa cells, the combination of BAA and DOX showed the CI was antagonistic in IC_12.5_ and synergistic in IC_25_, IC_50_, and IC_75_ at 24 and 72 h. Conversely, 48 h treatment resulted in synergism in IC_50_, but antagonism in IC_12.5_, IC_25_, and IC_75_. In HepG2 cells, a dose-reduction synergism appeared with BAA plus DOX in IC_12.5_, IC_25_, and IC_50_ concentrations at 24 and 48 h, while an antagonism effect was observed in IC_75_. An antagonism effect was found at 72 h in all concentrations (**Table 2**).

**Table 2:** Combination Index (CI) using the index-isobologram method developed by Chou and Talalay.

Cell line	Concentration	24 h	48 h	72 h
MCF-7	IC12.5	2.08 ± 0.38	1.35 ± 0.05	2.1 ± 0.3
	IC25	1.67 ± 0.34	0.74 ± 0.12	1.1 ± 0.1
	IC50	0.94 ± 0.17	0.5 ± 0.005	0.94 ± 0.1
	IC75	0.64 ± 0.29	1.84 ± 0.06	1.32 ± 0.4
MDA-MB	IC12.5	1.27 ± 0.6	2.8 ± 0.14	0.73 ± 0.03
	IC25	0.72 ± 0.17	2.35 ± 0.82	1.23 ± 0.32
	IC50	0.35 ± 0.07	1.75 ± 0.4	1.35 ± 0.03
	IC75	0.04 ± 0.01	0.52 ± 0.12	2.92 ± 0.48
HeLa	IC12.5	1.68 ± 0.53	1.28 ± 0.17	1.12 ± 0.38
	IC25	0.97 ± 0.17	1.17 ± 0.14	0.68 ± 0.05
	IC50	0.74 ± 0.16	0.93 ± 0.21	0.59 ± 0.02
	IC75	0.38 ± 0.25	1.16 ± 0.02	0.62 ± 0.29
HepG2	IC12.5	0.13 ± 0.07	0.19 ± 0.64	1.9 ± 0.1
	IC25	0.32 ± 0.1	0.41 ± 0.07	1.3 ± 0.3
	IC50	0.87 ± 0.05	0.55 ± 0.01	1.1 ± 0.2
	IC75	1.34 ± 0.31	1.53 ± 0.37	1.08 ± 0.2

*The CI equal to 1 indicates an additive effect; CI < 1 indicates synergy between the two drugs; and CI > 1 indicates antagonism between the two drugs.*

## Discussion

The aim of this study was to determine the cytotoxic effects of BAA against human breast, cervical, and liver cancer cells and its possible mechanisms of action. BAA significantly reduces cell proliferation in a concentration- and time-dependent manner. Based on the IC_50_ values of BAA, the extract’s potency was in the order of HepG2 > MCF7 > HeLa > MDA-MB-231 for BAA at three periods of time (24, 48, and 72 h). It should be taken into account that the metabolism of cells could differ from each other and an extract could possibly contain compounds which activate the apoptotic pathway signals in a cell that are more susceptible to be influenced by phytochemical compounds present in the extract, as compared to the other cells. An extract could also have contained compounds affecting different points in the apoptotic pathway which were more susceptible to influence. Moreover, these data revealed that BAA displays only selective inhibitory effects against cancer cells without affecting normal cells. Therefore, BAA could be a potential choice for the development of a cancer chemoprevention agent toward targeting breast, cervical, and liver cancers. Specific targeting of tumor cells serves to increase the therapeutic efficacy of anticancer drugs while at the same time reducing organ toxicities (Arafa, 2009). Although the active compounds of BAA are responsible for growth, the inhibitory effect has not been identified separately for each compound since a crude methanol extract was used, and the cytotoxic effect could be attributed to the crude extract itself.

The proportion of dead cells and type of cell death after treatment were better illustrated from the morphological observations. The observations showed that the morphological changes during cell death were consistent with cell apoptosis features. The extracts were more potent to induce early apoptosis (AO +, PI –) after 24 and 48 h and late apoptosis (AO +, PI +) after 72 h of treatment. However, MCF7 and MDA-MB-231 exhibited late apoptotic cells at 48 and 72 h. Necrotic (AO –, PI +) cell populations appeared very rarely. Early apoptotic cells attract phagocytes by the release of specific signals, without enhancement of inflammation, while late apoptotic and necrotic cells release additional pro-inflammatory danger signals (Ng et al., 2011; Liang et al., 2014). Interestingly, only a few necrotic cells were observed at 72 h of treatment. This suggested that the cell death might occur due to nutrient depletion in the growth media or contact inhibition.

Despite the fact that BAA inhibited the growth of the studied cancer cells, it blocked proliferation by inducing cell cycle arrest at sub-G0 peak in a dose-dependent manner for all cell lines. In contrast, the enhancement of cell population in the G0/G1 phase was observed only in HepG2 cells treated with lower concentrations of BAA. Moreover, the S phase and G2/M phase arrest of the cell cycle progressions were shown by the cell phase distribution in MCF7, MDA-MB-231 and HepG2, which were treated with lower doses of extract and exposed to the extracts for a shorter time. Deregulation of the cell cycle checkpoints could cause abnormal cell proliferation and cancer development (Chen et al., 2014). Progression through the S phase is controlled by monitoring replication checkpoints and moderates DNA synthesis. In the event of DNA damage, this checkpoint prevents cell-cycle progression (Dickson and Schwartz, 2009). Hence, it was possible that the increment of cells in S phase was due to the incorporation of BAA into a damaged DNA during the process of DNA replication. Moreover, the G2/M checkpoint prevented the damaged DNA from entering into mitosis (Sharon et al., 2005) and played an important role for DNA damage-induced apoptosis. Most anticancer drugs induced cancerous DNA damage, blocked mitosis and arrested cells at the G2/M phase (Li-Hua et al., 2012). Therefore, it can be understood that BAA prevents the occurrence of mitosis in the damaged cell, through arresting them in the G2/M phase. Overall, all these results suggested that extract-induced growth inhibitory effect is associated with the occurrence of apoptosis, more than cell cycle arrest. Thus, the apoptotic cell death of BAA was further analyzed.

Annexin V (a phagocyte membrane protein) which has a high affinity for phospholipid phosphatidylserine (PS) (Hawley and Hawley, 2004) and flow cytometry analysis showed a decline of viable cells percentage with the increasing concentrations of BAA in all the studied cell lines. The analysis revealed that the treatment with low and middle concentrations (IC_25_ and IC_50_) of BAA for 24 and 48 h resulted in more percentages of early apoptotic cells, while the treatment over a longer time (72 h) or with a high concentration (IC_75_) for 24, 48, and 72 h resulted in more cells present in the late apoptosis stage than early apoptosis. Early apoptotic cells can become late apoptotic cells when the plasma membrane becomes permeabilized (Poon et al., 2010). Treatment with BAA led to an increment of annexin V staining positive cells in a concentration-dependent manner. Only a small percentage of cells died via the necrotic pathway which could be because the cells dying by apoptosis were eventually degraded as necrotic cells due to losing their DNA repair ability at late apoptosis (Dartsch et al., 2002).

Apoptosis is executed by the cleavage of various caspases. Elucidating the consequence of caspase cleavage provides us with an insight into cell death and other biological processes (Shao et al., 2007). The caspase activity in treated MCF7 suggests that the increment of caspase-3, -8, and -9 activities in the same concentration (IC_50_) could deduct that both extrinsic and intrinsic pathways occurred to activate caspase-3 in this concentration. Caspase-mediated apoptosis could activate a death receptor (extrinsic) pathway or mitochondrial (intrinsic) pathway or both pathways together (Lim et al., 2014). The caspases activity in MDA-MB-231 illustrated that in different concentrations, various caspases are activated. In the IC_25_ of BAA, initiator caspase-8 resulted in the activation of caspase-3. In IC_50_ and IC_75_ of BAA, the executioner caspase -3 was not activated, while caspase-9 and caspase-8 were activated in at least one of these concentrations. Firstly, the results gave the possibility that apoptosis in MDA-MB-231 cells in these concentrations was not going through the caspase activation pathway, as many studies have shown that apoptosis is not always concomitant with the activation of caspases) Chiu et al., 2006). Secondly, possibly the other executioner caspase (caspase-7) was activated in these concentrations. Caspases-8 and -9 can activate both caspases-3 and -7 during death receptor- and DNA-damage-induced apoptosis, respectively. Caspase-3 and -7 have overlapping, but also distinct roles in apoptosis. However, the importance of these caspases is cell type- and stimulus-dependent (Lamkanfi and Kanneganti, 2010). In HeLa cells, caspase-9 and caspase-3 activation strongly indicated that a mitochondrial pathway is involved in the apoptosis induction by BAA in this cell line that produces intracellular signals to act directly on targets within the cell (Elmore, 2007).

The result of BAA treatment of HepG2 cells had not found any correlation between apoptosis induction and caspase activity, which demonstrated that induction of apoptosis might occur through other pathways.

In the current study, the effects of BAA on mRNA expression pattern of *Bcl-2*, *Cdk1* and *p53* were determined by the q-RT-PCR technique. One possible mechanism by which BAA induces apoptosis is thus through modulating the expression of the *Bcl-2* gene. *Bcl-2* antagonizes the effects of *BAX* (pro apoptotic gene) and prevents the release of cytochrome *c* (Portt et al., 2011). Alternatively, *Bcl-2* may be responsible for the loss of outer-membrane integrity and cytochrome *c* redistribution (Vander Heiden and Thompson, 1999). *Bcl-2* overexpression has been implicated in different carcinomas (Guo et al., 2014). In the current study, modulations in the expression of *Bcl-2* had been correlated with the type of cancer cells. Moreover, the data suggested that caspase-9-mediated disruption of the mitochondrion involved the cleavage of *Bcl-2*. In HeLa and HepG2, it has been observed that caspase-9 activity was aligned with *Bcl-2* expression, since the downregulation of *Bcl-2* potently enhanced caspase-9 activity in HeLa cells in all concentrations, while HepG2 exhibited a low activity of caspase-9.

Assessment of *Bcl-2* levels in the studied cells upon recovery from BAA illustrated some remarkable modulations, the nature of which appeared to be related to the exposure concentration of these agents. For example, the increment of BAA concentrations from IC_25_ to IC_75_ in MCF7 cells illustrated a downward trend of *Bcl-2* expression. The results were in agreement with caspase-9 activity which gradually increased after treatment with IC_75_ of BAA, compared to the control cells. Alternatively, MDA-MB-231 illustrated a remarkable downregulated *Bcl-2* in IC_25_ and IC_75_ of BAA, while caspase-9 activity increased only in IC_25_. It can be inferred that the caspase-9 pathway is not involved in the signal transduction pathways in MDA-MB-231 treated with other concentrations. Apparently, *Bcl-2* can regulate an independent pathway of mitochondrial cytochrome *c* release and caspase 9 activation which rather than initiate seems to amplify the caspase cascade (Marsden et al., 2002; Jiang and Milner, 2003). The variable expression of *Bcl-2* in cancer cells demonstrates a gene structure and/or expression abnormalities dependent regulation. Variations in the activation status of signal transduction pathways might result in variable expressions of the *Bcl-2*. The data indicated that upon treatment with BAA, the level of *Bcl-2* was downregulated in an altered fold and, depending on the cell line and concentration of extracts, supporting the downregulation of *Bcl-2* may be one of the molecular mechanisms through which BAA induced apoptosis and this could be one of the targets hit by BAA active compounds to induce apoptosis.

In addition, cancer can be described as a disease of genomes which occurs by dynamic changes of DNA in the cell cycle (Arıcan et al., 2014). *Cdk1* controls cell cycle entry from G2 to the M phase (Chen et al., 2015) and is considered as a new anti-cancer drugs target (Guo et al., 2015). Different doses of drug may activate several regulatory mechanisms, resulting in either apoptosis or cell death. Results of the cell cycle arrest indicated that a high dose of BAA induced apoptosis, whereas *Cdk1* was significantly downregulated; in contrast, in a low dose, the extracts induced cell death through mitotic catastrophe, while *Cdk1* was slightly downregulated. Moreover, the cell cycle phases of HeLa cells were not altered by BAA, whereas the expression of *Cdk1* was downregulated by BAA. These observations could be explained regarding the fact that the activation of a *Cdk1* requires binding to a specific Cyclin (Cyclin B). This complex controls the timing of mitotic entry and cell division. Lake of Cyclin B synthesis before G2/M transition results an inactive *Cdk1.* Therefore, the cell cycle will arrest at the G2 phase (Liu et al., 2008). Thus, maybe the expression of Cyclin B was reduced in the lower concentrations of the extracts. In addition, *Cdk1* activation is a necessity for entry into apoptosis. It can stimulate the apoptotic pathway activation in a direct way, through an action of *Bcl-2* family proteins. Hence, the down regulation of *Cdk1* in a higher dose induced apoptosis; however, the cells in the G2 phase were not altered significantly. Since the role of *Cdkl* in the control of apoptosis may depend on the cytotoxic stimuli and cellular context, a cell type-specific modulation of *Cdk1* might be taken advantage of the therapeutic correction of pathogenic imbalances in apoptosis control (Castedo et al., 2002; Park et al., 2005).

The tumor suppressor gene *p53*, prevents tumorigenesis in response to physiological and environmental stress and is mutated in over 50% of human tumors (Gupta et al., 2001). In the current study, BAA exhibited a downregulation effect on MCF7 and HeLa cells (significant in IC_25_), while it upregulated considerably in MDA-MB-231 treated with IC_25_ and IC_75_. The same phenomena occurred for HepG2 in all of the concentrations. The *p53* gene negatively regulates *Cdk1* and cyclin B1 transcription (Castedo et al., 2002). In fact, up-regulation of *p21* by *p53* resulted in decreasing the Cyclin B1/*Cdk1* complex (Gupta et al., 2001) Treatment with IC_75_ of BAA in MDA-MB-231 and all concentrations of BAA in HepG2 may follow this pathway, as the upregulation of *p53* was accompanied with the downregulation of *Cdk1*. However, the expression of *p53* was kept unchanged or decreased in some concentrations of BAA after treatment, while *Cdk1* expression was reduced. It was supposed that inducing the cell cycle arrest and apoptosis without *p53*/*p21* system being activated and downregulating the level of *Cdk1* were independent of *p53*. The current study did not exclude the possibility that some of the cells that were negative for nuclear *p53* expression may have *p53* gene mutation (Arıcan et al., 2014). Therefore, the results suggested that the induction of apoptosis by extracts in most concentrations on the cell lines had downregulated the expression of dysfunctional *p53* and the induction of apoptosis was independent of *p53* status.

Combination chemotherapy is a more efficient method in the treatment of cancer, compared to single-agent treatment. The current study indicated a considerable dose reduction when the herbal extracts and commercial drugs were used together. While TAM alone decreased MCF7 cell growth, adding BAA extract to TAM resulted in a remarkable dose- and time-dependent inhibition. Such a combined effect was significant 1–2 times higher than those of TAM alone. Interestingly, a similar phenomenon occurred in the presence of the MDA-MB-231 cells, which were responsive to TAM alone and in the combination with BAA. It is not clear whether this combined cytotoxicity is an estrogenic receptor-dependent mechanism, as the MDA-MB-231 is known as being estrogen receptor-negative (Al-Akoum et al., 2007). This combined effect is probably because of the drugs with different mechanisms of action, proposing that the combination drug could act in a multifactorial pathway (Yoshimaru et al., 2013). The other chemotherapy drug, DOX, acts by inhibiting DNA replication and mitosis (Chang et al., 2006). DOX can inhibit the synthesis of both DNA and RNA. In addition, DOX exerts its effects via preventing the activity of uncoiling of the DNA enzyme, topoisomerase II. However, increased risk of bleeding and infection, loss of appetite, cardiac damage and heart failure are the inevitable side effects (Wong et al., 2013). It could be suggested that the mechanism of action of BAA is different from the pathway that DOX imposes its effect, where this difference allows the combination drug to attack along multiple pathways to prevent cancer cell resistance and accelerate the treatment process. Moreover, overlapping toxicities may be restricted by different mechanisms. In addition, the different metabolic responses between the extracts and commercial drugs (TAM and DOX) might, in part, contribute to the synergistic effect of these two agents and minimize TAM and DOX side effects by reducing the dosage. For all of the cell lines, the required dose for combined drugs was lower than their IC_50_ alone which provided evidence supporting the use of BAA in combination with chemotherapy drugs. It is, therefore, worthwhile to clinically examine the mechanisms of this synergism.

## Conclusion

BAA shows anti-cancer effects on MCF-7, MDA-MB-231, HeLa, and HepG2 cells in different dosage. The effect was mediated through the inhibition of cell proliferation of the cancer cells. The underlying mechanisms involved the stimulation of cell-specific G0/G1, S, and G2/M cell cycle arrest and the induction of apoptosis through both extrinsic and intrinsic apoptotic pathways. In addition, the BAA -induced apoptosis in MCF-7 and HeLa cells is likely to be caspase-dependent and not p53 transcription-dependent align with downregulation of *Bcl-2* and *Cdk1*, while it is *p53*-dependent in MDA-MB-231 and HepG2. BAA also exhibited synergism when combined with TAM and DOX, suggesting that it can contribute with current chemotherapeutic agents. This study supports the hypothesis that BAA can potentially be used in certain anti-cancer therapy and thus paving the way for further research on BAA in the field of anti-cancer drug discovery.

## Author Contributions

The corresponding author, PI, has generated the idea for the experiment and has critically examined and corrected the manuscript. SK, the first author has conducted the experimental work and written the manuscript. Other authors, RH, NE, VR, and AP, have helped during the experimental work and in the interpretation of the results. All authors read and approved the final manuscript.

## Conflict of Interest Statement

The authors declare that the research was conducted in the absence of any commercial or financial relationships that could be construed as a potential conflict of interest.
